# Deep learning single-cell analysis for cytologic evaluation of oral potentially malignant disorders

**DOI:** 10.1038/s41598-026-47538-y

**Published:** 2026-07-13

**Authors:** Michael P. McRae, Kritika S. Rajsri, Nadarajah Vigneswaran, A. Ross Kerr, Spencer W. Redding, Martin H. Thornhill, Craig Murdoch, Paul M. Speight, Nancy Ruel, Rachelle Wolk, Ryan R. Ruff, John T. McDevitt

**Affiliations:** 1 Custom DX Solutions LLC, Houston, TX US; 2 Department of Molecular Pathobiology, Division of Biomaterials, Bioengineering Institute, New York University College of Dentistry, New York, NY US; 3 Department of Diagnostic and Biomedical Sciences, The University of Texas Health Science Center at Houston, Houston, TX US; 4 Department of Oral and Maxillofacial Pathology, Radiology & Medicine, New York University College of Dentistry, New York, NY US; 5 Department of Comprehensive Dentistry and Mays Cancer Center, The University of Texas at San Antonio Health Science Center, San Antonio, TX US; 6 Department of Oral & Maxillofacial Medicine, Surgery and Pathology, School of Clinical Dentistry, University of Sheffield, Sheffield, UK; 7 Department of Epidemiology and Health Promotion, New York University College of Dentistry, New York, NY US; 8 Department of Molecular Pathobiology, New York University College of Dentistry, 345 East 24th Street, 8th Floor Room 808, New York, NY 10010 USA

**Keywords:** Oral potentially malignant disorders, Oral epithelial dysplasia, Oral squamous cell carcinoma, Deep learning, Artificial intelligence, Intelligent cytology microfluidics, Cancer, Diseases, Medical research, Oncology

## Abstract

Oral potentially malignant disorders (OPMDs) such as leukoplakia and erythroplakia may harbor dysplasia or progress to oral squamous cell carcinoma (OSCC), yet visual inspection alone is unreliable for risk assessment. Cytology offers a minimally invasive adjunct, but conventional approaches depend on manual feature extraction and subjective review. Herein we report a deep learning (DL) object detection model that directly classifies four cell phenotypes: differentiated squamous epithelial (DSE) cells, small round (SR) cells, leukocytes, and lone nuclei. The DL model produced cytology-derived parameters that correlated strongly with histopathologic diagnoses across 692 subjects with OPMDs, OSCC, and healthy controls, including declining DSE cell proportion and increasing SR cells and leukocytes with disease severity (*p* < 0.0001). The oral cancer numerical index (OCNI) achieved AUROC values up to 0.99 for malignant versus healthy lesions, with excellent reliability (intra-class correlation coefficient ≥ 0.96). This reproducible, minimally invasive test provides a robust platform for early detection and surveillance of OPMDs.

**Supplementary Information:**

The online version contains supplementary material available at 10.1038/s41598-026-47538-y.

## Introduction

Oral potentially malignant disorders (OPMDs) such as leukoplakia and erythroplakia, among others, can harbor oral epithelial dysplasia (OED) or oral squamous cell carcinoma (OSCC), yet visual inspection alone is insufficient for reliable risk assessment^1,2^. Oral cytology is a minimally invasive adjunct that has been demonstrated to correlate with histopathology^3–5^; however, conventional approaches depend on remote processing and subjective interpretation, delaying results and limiting point-of-care (POC) use.

Cytological signatures are morphological, phenotypical, and molecular features of individual cells that offer objective diagnostic information. Previously, analysis of cytological images relied on feature extraction followed by machine learning (ML) to identify the composition of cell phenotypes in a given sample^6^. However, this approach lacked reliability across imaging platforms and was sensitive to changes in imaging conditions. Deep learning (DL) object detection networks now enable automated localization and classification of cells directly from cytology images, bypassing error-prone feature extraction methods^7^.

Herein we report the development and validation of an intelligent cytology microfluidics (Cyt-MF) system for automated cell phenotype identification in cytological samples from subjects with OPMDs, OSCC, and healthy controls. Trained on a large, clinically annotated oral cytology-on-a-chip dataset^8,9^, the DL model detects and classifies individual cells, enabling rapid, reproducible phenotyping. We compared the reliability of cytology measurements derived from this DL method to an earlier analysis version which relied on conventional feature extraction and ML^10,11^. This Cyt-MF system has the potential to advance triage of patients with OPMDs, and surveillance of patients with OED or OSCC, by delivering expert-level cytology analysis on a POC-compatible platform.

## Results

### Subject characteristics

**Fig. 1:**
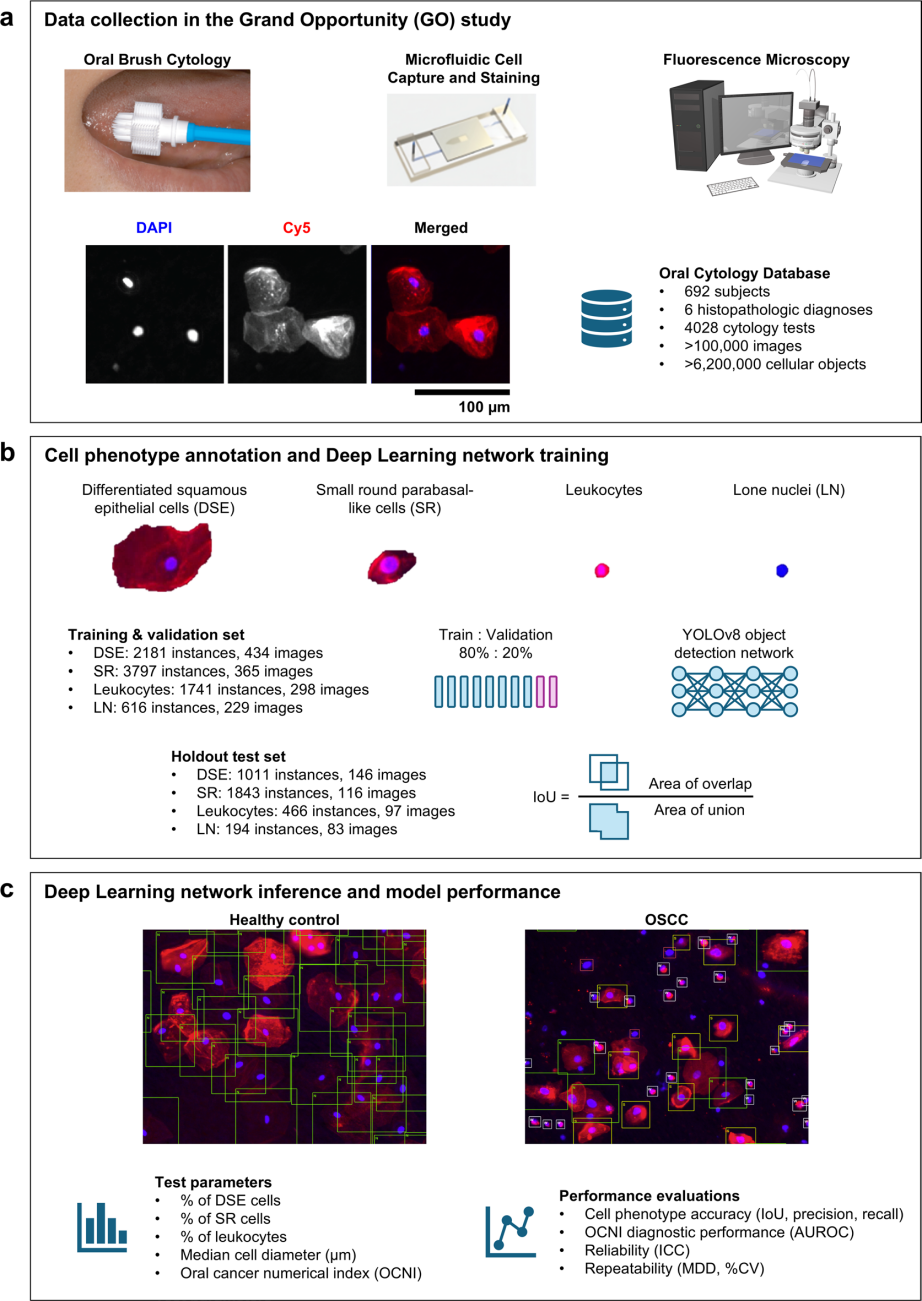
Overview of the development of the Deep Learning (DL) model for the Cyt-MF platform. (**a**) Data collection in the Grand Opportunity (GO) study involved collection of brush cytology specimens from subjects with oral potentially malignant disorders (OPMDs), previously diagnosed oral squamous cell carcinoma (OSCC), and healthy controls, completion of cytological analysis via a microfluidic cell capture and fluorescence microscopy, and generation of one of the largest oral cytology databases with paired histopathologic diagnoses. (**b**) Development of the DL model for single-cell phenotype classification involved annotation of four cellular phenotypes, training of an object detection network, and evaluation of the model performance on a holdout test set by bounding box localization, precision, and recall. (**c**) The DL model was evaluated on cytology images from the GO study spanning six histopathological diagnoses and healthy controls, and performance of the test parameters was evaluated for individual cell classification accuracy, diagnostic accuracy, reliability, and repeatability. Images from panel A were reprinted from Abram et al. (2019), Development of a cytology-based multivariate analytical risk index for oral cancer, doi: 10.1016/j.oraloncology.2019.02.011, with permission from Elsevier.

The data used in this study originated from the 1053-patient Grand Opportunity (GO) study which aimed to assess the diagnostic accuracy of a cytology-on-a-chip system relative to scalpel biopsy and histopathology^8–11^. Fig. 1a summarizes the data collection in the GO study which involved collection of brush cytology specimens from subjects with OPMDs, previously diagnosed OSCC, and healthy controls; completion of cytological analysis via a microfluidic cell capture and fluorescence microscopy; and, generation of one of the largest oral cytology databases with paired histopathologic diagnoses, representing over 100,000 images and > 6.2 million cellular objects.

Out of the 1053 subjects enrolled in the GO study, 54 withdrew, and 307 were excluded from the analysis for the following reasons as summarized in **Figure ****S1**: partial cytology measurements (*n* = 21); inadequate number of cells in the sample (*n* = 47); sample used in assay/process development (*n* = 2); samples lost due to shipping/freezer failure (*n* = 44); and cytology not measured due to funding constraints (*n*= 193). A total of 692 subjects were included in this analysis: 144 healthy control, 325 benign, 65 mild dysplasia, 27 moderate dysplasia, 17 severe dysplasia, and 114 malignant. Previously, we reported results for a total of 486 subjects in which image data for 206 subjects were collected but not analyzed^10,11^. In this current analysis, the images for those 206 subjects were included for either training or test set evaluation.

Subjects with OED (mild, moderate, severe, or carcinoma in situ) or OSCC were significantly older than those with benign lesions (59 ± 13 vs. 56 ± 14 years, *p* = 0.0045) and more often male (57% vs. 46%, *p* = 0.0166) (Table 1). Hispanic ethnicity was less common among benign cases compared with OED/OSCC (*p* = 0.0205). Alcohol consumption frequency (days/year) was higher in subjects with OED/OSCC (*p* = 0.0077). Tobacco use was more prevalent in subjects with OED/OSCC for any history (71% vs. 56%), former smoking (68% vs. 52%), and current smoking (36% vs. 20%) (all *p* < 0.001).

**Table 1:** Subject characteristics and cytology test parameters.

	Controls (*n* = 144)	Benign (*n* = 325)	OED & OSCC (*n* = 223)	*p* value ^a^
Age (years)	45.8 ± 15.6	55.8 ± 14.0	59.2 ± 12.9	**0.0045**
Male	42 (29.2%)	150 (46.2%)	127 (57.0%)	**0.0166**
Race				
White	129 (89.6%)	273 (84.0%)	185 (83.0%)	0.8371
Black or African American	8 (5.6%)	22 (6.8%)	20 (9.0%)	0.4311
Asian	6 (4.2%)	24 (7.4%)	16 (7.2%)	1.0000
American Indian or Alaskan Native	3 (2.1%)	0 (0.0%)	2 (0.9%)	0.3225
Other	2 (1.4%)	7 (2.2%)	4 (1.8%)	1.0000
Ethnicity				
Hispanic	60 (41.7%)	43 (13.2%)	47 (21.1%)	**0.0205**
Alcohol				
Former drinker	128 (88.9%)	290 (89.2%)	200 (89.7%)	0.9770
Current drinker	82 (56.9%)	211 (64.9%)	139 (62.3%)	0.5962
Days per year	52.9 ± 84.4	84.9 ± 114.3	114.3 ± 141.9	**0.0077**
Average drinks per day	2.2 ± 3.0	2.1 ± 2.4	2.1 ± 2.4	0.9891
Tobacco				
Any tobacco history	66 (45.8%)	181 (55.7%)	159 (71.3%)	**0.0003**
Former smoker	62 (43.1%)	169 (52.0%)	151 (67.7%)	**0.0003**
Current smoker	28 (19.4%)	64 (19.7%)	81 (36.3%)	**< 0.0001**
Smoking pack-years	6.1 ± 12.8	9.7 ± 17.3	19.2 ± 26.0	**< 0.0001**
Lesion characteristics				
Size				
Major axis length (mm)	-	15.4 ± 11.2	22.5 ± 15.4	**< 0.0001**
Minor axis length (mm)	-	9.9 ± 7.8	15.7 ± 12.4	**< 0.0001**
Area (mm^2^)	-	684.6 ± 1134.7	1624.5 ± 2418.6	**< 0.0001**
Diffuse or unable to measure	-	46 (14.2%)	19 (8.5%)	0.0616
Color				
White	-	133 (40.9%)	69 (30.9%)	**< 0.0001**
Red	-	59 (18.2%)	38 (17.0%)	0.8246
Red and white	-	133 (40.9%)	115 (51.6%)	**< 0.0001**
Appearance				
Patch/plaque	-	229 (70.5%)	121 (54.3%)	**< 0.0001**
Nodule/mass	-	68 (20.9%)	75 (33.6%)	**< 0.0001**
Ulcer	-	31 (9.5%)	71 (31.8%)	**< 0.0001**
Erosive	-	26 (8.0%)	26 (11.7%)	0.1979
Multiple lesions	-	151 (46.5%)	67 (30.0%)	**< 0.0001**
Cytology test parameters				
DSE Cells (%)	95.8 ± 2.8	82.7 ± 18.5	61.1 ± 30.9	**< 0.0001**
SR Cells (%)	3.3 ± 2.3	9.8 ± 9.7	18.4 ± 13.6	**< 0.0001**
Leukocytes (%)	0.9 ± 0.7	7.5 ± 14.7	20.4 ± 25.9	**< 0.0001**
Lone nuclei (%)	17.8 ± 14.2	23.8 ± 16.0	18.7 ± 14.2	**0.0001**
Median cell diameter (µm)	72.4 ± 3.6	67.7 ± 8.1	59.6 ± 12.3	**< 0.0001**
OCNI	16.3 ± 7.4	31.4 ± 17.0	54.4 ± 23.7	**< 0.0001**

^a^p values for comparison of benign versus OED & OSCC, continuous data by independent two-sample t-test and proportions by chi-squared test.

Abbreviations: OED, oral epithelial dysplasia (mild, moderate, severe and carcinoma in situ); OSCC, oral squamous cell carcinoma; DSE, differentiated squamous epithelial cells; SR, small round parabasal-like cells; OCNI, oral cancer numerical index.

Compared with benign lesions, OED/OSCC lesions were larger, less often white (31% vs. 41%), more often mixed red and white (52% vs. 41%), and more frequently presented as nodules/masses (34% vs. 21%) or ulcers (32% vs. 10%), but less often as patches/plaques (54% vs. 71%) or presenting with multiple lesions (30% vs. 47%) (all *p* < 0.0001).

### Cytology test parameters

Figure 1b shows examples of the cell phenotypes annotated for the DL model and their respective abbreviations within the annotated images: differentiated squamous epithelial (DSE) cells (denoted ‘N’), small round (SR) parabasal-like cells (denoted ‘S’), leukocytes (denoted ‘W’), and lone nuclei (LN) (denoted ‘L’). Figure 1c displays a small portion of a representative image of a healthy control sample with the DL model’s predictions for localization (bounding box) and cell phenotype labels overlaid showing primarily DSE cells (left image) and a representative image from a subject with OSCC with a substantially higher percentage of SR cells and leukocytes (right image).

Compared with benign lesions, OED/OSCC lesions samples had significantly lower proportions of DSE cells (61% vs. 83%) and lone nuclei (19% vs. 24%), and significantly higher proportions of SR cells (18% vs. 10%) and leukocytes (20% vs. 8%) (all *p* < 0.0001) (Table 1). The median cell diameter reflected the phenotypic composition of the cytology sample, with significantly smaller mean diameter in OED/OSCC versus benign lesions (60 μm vs. 68 μm) (*p*< 0.0001). Values of the oral cancer numerical index (OCNI)^10,11^, a score from 0 to 100 that represents the probability of OED or OSCC (predictors included age, sex, tobacco history, lesion color, lesion size, lesion appearance, presence of multiple lesions, DSE cells, and SR cells), were significantly higher for OED/OSCC lesions than those for benign lesions (54 vs. 31) (*p* < 0.0001). Interestingly, cytology parameters also showed highly significant differences between benign lesions and healthy controls (all *p* < 0.0001).

### Accuracy of cell phenotype identification

**Fig. 2:**
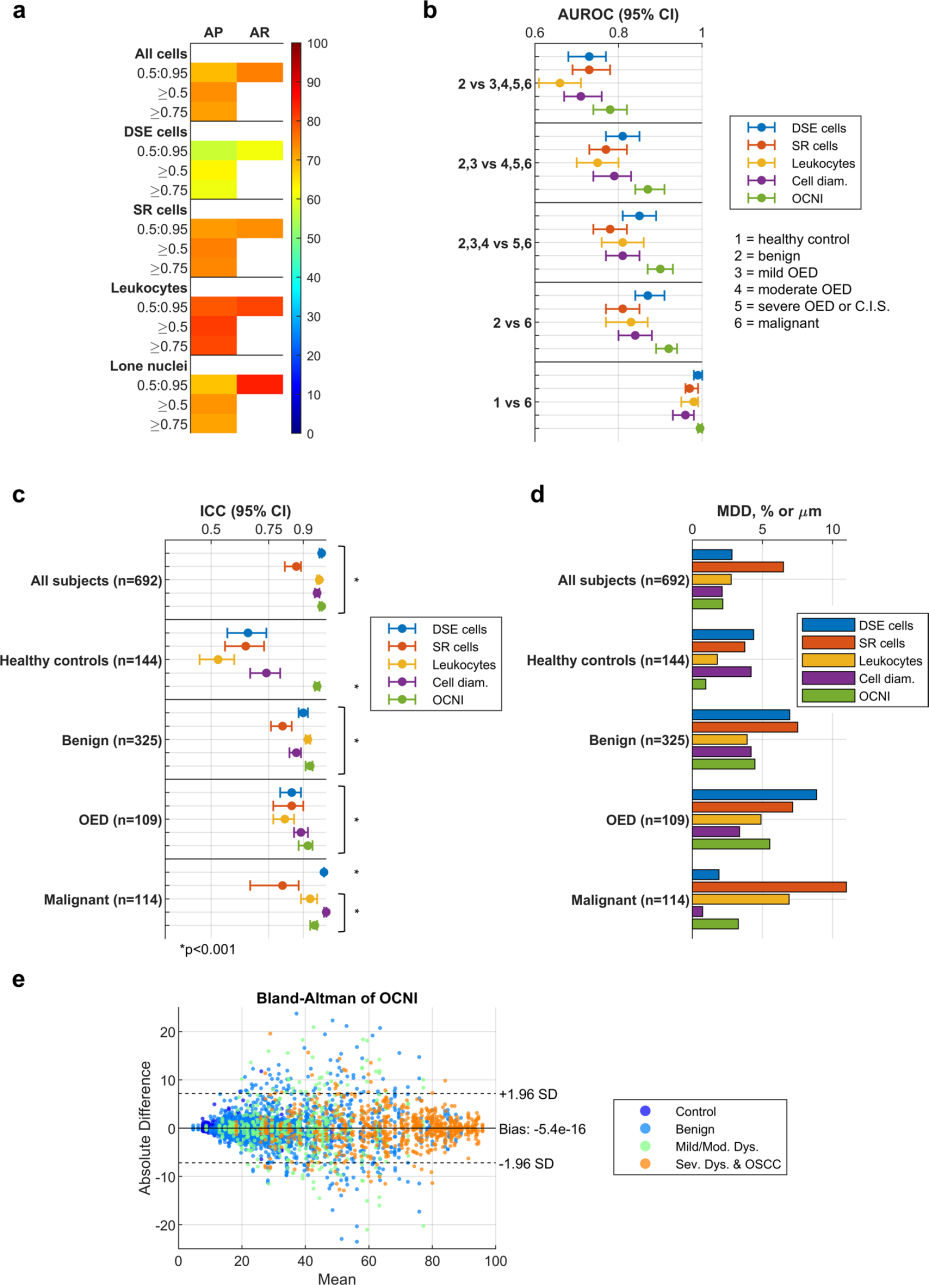
Performance evaluation of the Deep Learning model for the Cyt-MF platform: (**a**) average precision (AP) and average recall (AR) for identifying cell phenotypes at various intersection over union (IoU) criteria; (**b**) diagnostic performance of cytology test parameters relative to histopathology by area under the receiver operating characteristic (AUROC); within-sample reliability (**c**) by intra-class correlation coefficient (ICC) and repeatability (**d**) by minimum detectable difference (MDD) of the cytological assay across all subjects, and by histopathologic diagnosis; (**e**) Bland-Altman analysis of repeated OCNI measurements in subjects with OPMDs, OSCC, and healthy controls, representing 692 subjects and 4028 tests. Abbreviations: C.I.S., carcinoma in situ; DSE, differentiated squamous epithelial cells; SR, small round parabasal-like cells; OCNI, oral cancer numerical index; OED, oral epithelial dysplasia.

On the independent test set, the DL model achieved a mean Average Precision (mAP) of 69.0% and an average recall of 74.7% for all cell phenotypes at intersection over union (IoU) of 0.5:0.95, with performance increasing to 73.1% mAP at IoU ≥ 0.5 (Fig. 2a and **Table ****S1**). Among individual phenotypes, leukocytes showed the highest precision (78.8%) and recall (80.5%), followed by SR cells (precision 72.0%, recall 73.1%) and lone nuclei (precision 68.2%, recall 84.0%). The DSE cells were the most challenging class, with precision of 57.2% and recall of 61.2%.

### Diagnostic accuracy relative to histopathology

The diagnostic performance of cytology parameters is shown in Fig. 2b and **Table S2**. Among individual phenotypes, DSE cells also performed strongly, ranging from an AUROC of 0.73 for benign vs. mild–malignant to 0.99 for control vs. malignant. Leukocytes generally showed the lowest performance, with AUROCs ranging from 0.66 for benign vs. mild–malignant to 0.98 for control vs. malignant.

Across all histopathological diagnoses, OCNI consistently demonstrated the highest diagnostic performance. For benign vs. mild OED–malignant classifications, OCNI achieved an AUROC of 0.78, increasing to 0.87 for benign–mild OED vs. moderate OED–malignant, 0.90 for benign–moderate OED vs. severe OED–malignant, 0.92 for benign vs. malignant, and 0.99 for control vs. malignant.

**Fig. 3:**
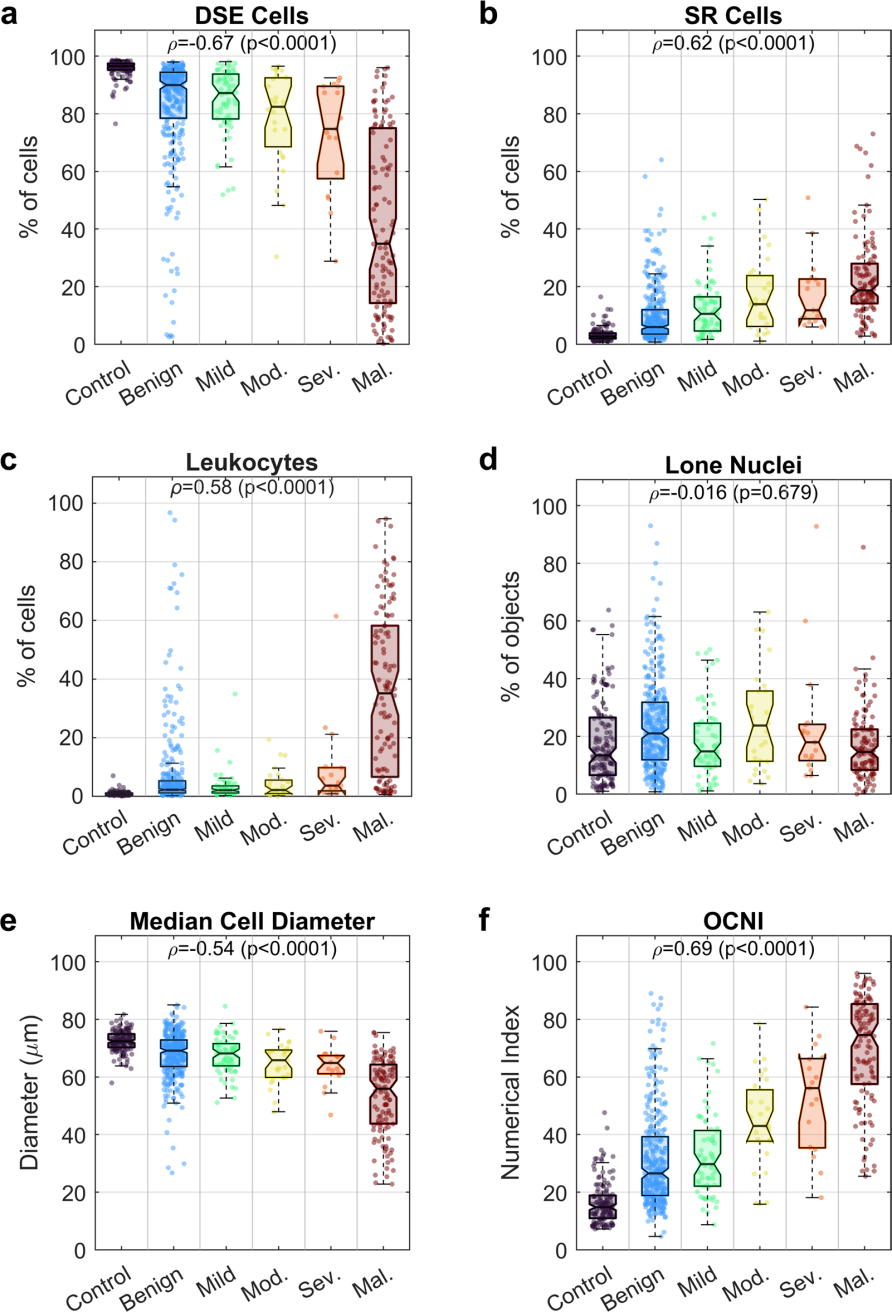
Distribution of cytology test parameters in subjects with OPMDs, OSCC, and healthy controls (*n* = 692): (**a**) DSE cells, (**b**) SR cells, (**c**) leukocytes, (**d**) lone nuclei, (**e**) median cell diameter, and (**f**) OCNI. Abbreviations: Mod., moderate dysplasia; Sev., severe dysplasia & carcinoma in situ; Mal., malignant; DSE, differentiated squamous epithelial cells; SR, small round parabasal-like cells; OCNI, oral cancer numerical index.

Scatter boxplots of cytology test parameters derived from the DL model across histopathologic diagnoses (benign, mild, moderate, and severe dysplasia, and malignant) and healthy controls are shown in Fig. 3 (median and interquartile ranges in **Table S3**). Several parameters demonstrated strong monotonic relationships with increasing disease severity. The proportion of DSE cells (Spearman ρ = − 0.67) and median cell diameter (ρ = − 0.54) decreased significantly with increasing disease severity, while SR cells (ρ = 0.62), leukocytes (ρ = 0.58), and OCNI (ρ = 0.69) increased significantly with disease severity (all *p* < 0.0001). Consistent with these correlations, Kruskal-Wallis tests revealed highly significant differences across histopathologic diagnoses and controls for all parameters (*p* < 0.0001), highlighting their discriminatory potential in distinguishing progressive cytological differences across the spectrum of disease severity. The presence of lone nuclei (ρ = − 0.016, *p* = 0.679) did not correlate significantly with disease severity.

### Within-sample reliability and repeatability

Up to six repeat cytology measurements were conducted across the 692 subjects, representing a total of 4028 repeat tests (**Table S4**). Among all subjects, within-sample reliability was high for all cell phenotype measurements, with reliability by intra-class correlation coefficient (ICC) ranging from 0.87 to 0.98, all exceeding the 0.75 threshold (all *p* < 0.0001) (Fig. 2c and **Table S5**). The values of OCNI, DSE cells, leukocytes, and median cell diameter each showed excellent reliability (ICCs ≥ 0.96), while SR cells demonstrated good reliability (ICC 0.87).

In terms of repeatability, DSE cells, median cell diameter, and OCNI demonstrated low variability (%CV ≤ 8.2%) and small minimum detectable differences (MDD) (Fig. 2d). The values of SR cells and leukocytes were more variable (%CV 36%–47%) with larger MDDs relative to the mean values across all subjects, due in part to the relatively low mean proportions found in any given sample. Bland-Altman analysis of the repeated OCNI measurements revealed a mean bias of 0 and limits of agreement (± 1.96 SD) of ± 7.2 (Fig. 2e).

Stratification by histopathology revealed strong reproducibility of test parameters across benign, OED, and malignant groups. Overall, DSE cells, median diameter, and OCNI consistently provided the most reliable and repeatable measures across diagnostic categories, whereas SR cells and leukocytes were more variable, again due to low baseline proportions. Healthy controls showed excellent repeatability for DSE cells, median cell diameter, and OCNI (%CV ≤ 5.3%); however, reliability was lower for cell-based measurements (ICC 0.53–0.66). This reduction in reliability is due to the lower biological heterogeneity within the healthy group, since ICC is defined as the ratio of between-subject variance to the total variance.

Compared with the DL-based method, the feature extraction and ML approach had substantially higher variability and worse reliability for every test parameter (**Table S6**). The only parameter for which ML reliability approached DL reliability was OCNI (ICC 0.72–0.92).

### Evaluation of the point-of-care Cyt-MF platform

**Fig. 4:**
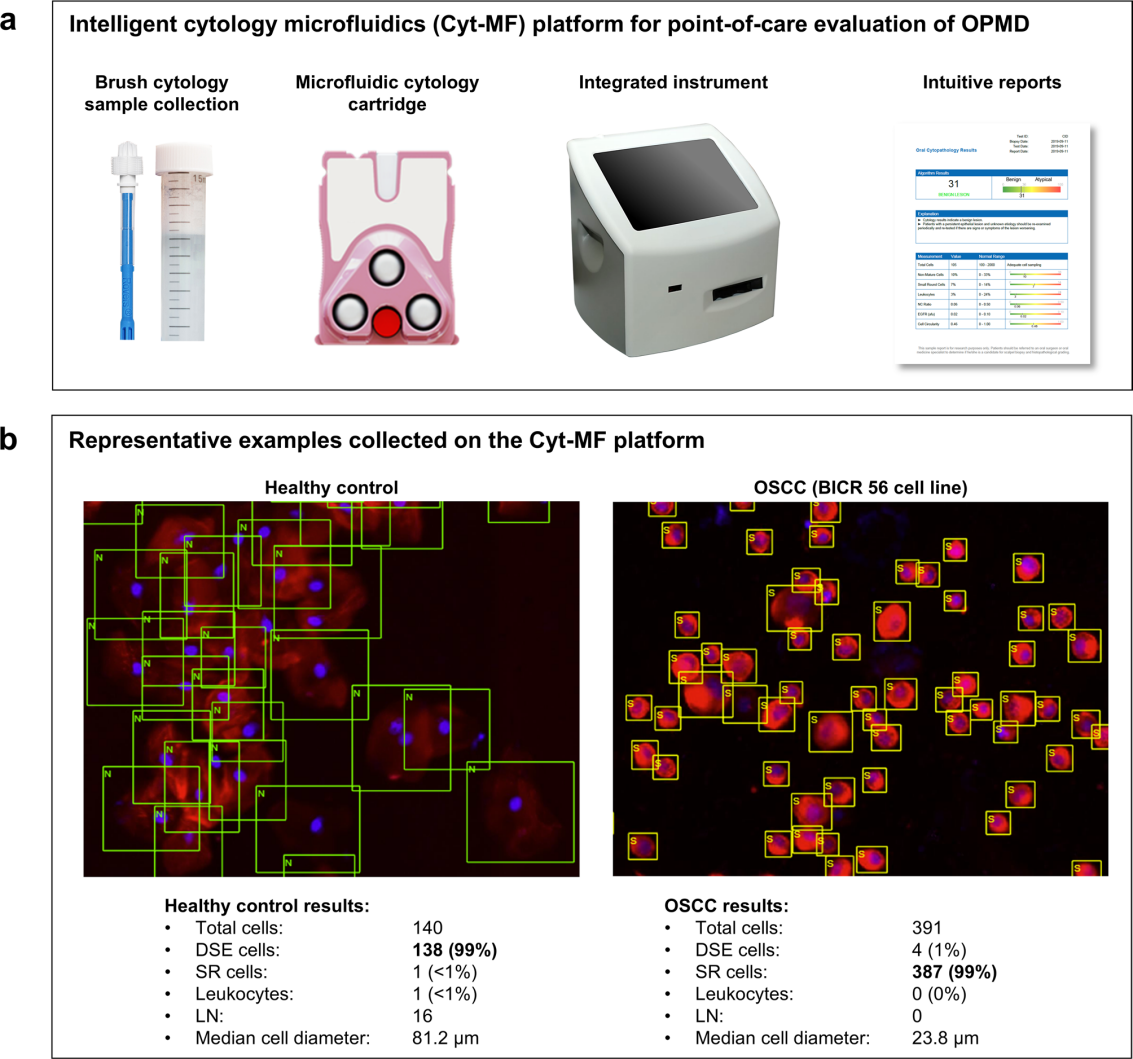
Evaluation of the Deep Learning cell phenotype model on the point-of-care (POC) Cyt-MF platform. (**a**) The Cyt-MF platform enables POC cytology measurements via convenient brush cytology sample collection, microfluidic cartridges with embedded reagents, integrated instrument with automated fluid delivery and multispectral fluorescence imaging, and intuitive reporting of the cytology test parameters. (**b**) Proof of concept of the integrated POC Cyt-MF platform comparing cytology results from a healthy control sample (left) and a sample with OSCC cells (BICR 56 cell line) (right). Images from panel A were reprinted from Rajsri et al. (2024), A brief review of cytology in dentistry, doi: 10.1038/s41415-024-7075-7, with permission from Springer Nature.

The POC Cyt-MF platform^10^(Fig. 4a) comprises a convenient brush cytology test kit, single-use microfluidic cartridge, integrated instrument, automated analysis of cytology test parameters, and intuitive reporting of results. To demonstrate the basic functionality of the POC Cyt-MF platform, we analysed two samples representing extreme phenotypes of healthy control and OSCC cells (BICR 56 cell line) (Fig. 4b). The healthy control sample would be expected to be primarily comprised of DSE cells and the OSCC cell line would be expected to be homogeneously composed of SR cells. In the healthy control sample, the DL model identified 138 (99%) DSE cells, 1 (< 1%) SR cell, and 1 (< 1%) leukocyte. The median cell diameter of 81.2 μm reflected the high proportion of DSE cells. In contrast, in the sample with OSCC cells, the DL model identified 387 (99%) SR cells, 4 (1%) DSE cells, and no leukocytes. The median cell diameter of 23.8 μm reflected the high proportion of SR cells. These preliminary results verify that the POC Cyt-MF platform correctly classified the expected cell phenotypes in the respective cytology samples and support the need for additional validation in the intended use population.

## Discussion

In this study of subjects with OPMDs, prior OSCC, and healthy controls, we demonstrated that an intelligent cytology microfluidics (Cyt-MF) system can reliably quantify cell phenotypes and generate diagnostic parameters that reflect histopathologic severity. The DL model achieved good detection accuracy (mAP 69%, recall 75%). Cytologic differences between histopathologic diagnoses were strongly monotonic with increasing disease severity, supporting their biological relevance and diagnostic potential. OCNI values consistently showed strong diagnostic performance, with AUROCs up to 0.99 for distinguishing healthy controls versus malignant lesions. Test parameters quantified by the DL method, including OCNI, DSE cells, and cell diameter, had excellent reliability (ICC 0.96–0.98) and repeatability (%CV 3.4%–8.2%) and outperformed the conventional feature extraction and ML approach (ICC 0.74–0.92 and %CV 12.9%–20.6%). Together, these findings demonstrate that the Cyt-MF system provides a robust tool for risk stratification of patients with OPMDs, with the potential to reduce unnecessary biopsies in patients with low risk and increase the yield of referrals for patients with higher risk for OED and OSCC.

Previously, we reported an oral cytology approach comprising microfluidics, multispectral fluorescence imaging, and single-cell analytics^12^. A prospective validation study developed one of the largest oral cytology databases for OPMDs^9 ^in which brush cytology measurements were correlated with six levels of histopathological diagnosis^8^. Diagnostic accuracy for the cytology-on-a-chip^11^, using conventional feature extraction and ML methods, rivaled and exceeded that of commercially available adjuncts^13–16^. The development of a convenient brush cytology sample collection kit, single-use microfluidic cartridges, and integrated instrumentation enabled cytology assays at POC^10^. The DL-based cell phenotype classifier further improves this POC oral cytopathology tool, now referred to as the intelligent Cyt-MF system, towards expert-level stratification of risk for OED and OSCC, compatible with POC assessments of oral lesions in primary care settings.

Recent important advances in artificial intelligence have demonstrated the feasibility of applying DL to oral cytology images^17^, including a convolutional neural network (CNN)–based classifier that can distinguish diagnoses in exfoliative cytology slide preparations^18,19^, semantic segmentation model allowing the identification of individual epithelial cells in multi-spectral fluorescence images^20^, and automated nucleus detection using a sliding-window and Mask-RCNN approach^21,22^. Collectively, these studies show strong interest in applying DL methods to the cytologic evaluation of patients with OPMDs.

Previously, several large oral cytology studies have been completed (Folsom et al., with 6879 subjects; Deuerling et al., with 1352 specimens from 992 subjects; and Sciubba, with 945 subjects)^14,23,24^. Several aspects of the current work are unique. The GO study was an international, multi-site, prospective study of 692 subjects representing the spectrum of patients with OPMDs, prior OSCC, and healthy controls. All cytology test results in subjects with OPMDs and OSCC were supported by histopathologic diagnoses using a two- or three-stage pathology adjudication process. Repeated measurements, up to six per sample for a total of 4028 tests, allowed evaluation of reliability and repeatability across the spectrum of disease. The DL-based evaluation of cytology images resulted in the detection of over 6.2 million cellular objects in over 100,000 images, representing a significantly larger dataset than other DL models for OPMDs diagnosis and prognosis^17^.

One challenge in model development is determining the number of instances and images to include in training and validation. Transfer learning is a training approach that uses knowledge generated from a previous classification task and modifies the network to accomplish a different but similar task^25^. As a result of leveraging prior knowledge in image classification tasks, significantly less labeled data are required to train the network, and the computational complexity of the learning task is significantly reduced. Commonly cited rules of thumb state that only 50–100 examples per class are needed for proof-of-concept level performance, 200–500 for good performance, and > 1,000 for excellent performance^26,27^. In our training set, we exceeded the recommended examples per class for excellent performance (> 2,100 DSE cells, > 3,500 SR cells, and > 1,700 leukocytes).

A COCO-pretrained model (YOLOv8) and transfer-learning framework was used because it offered strong detection performance, efficient training/inference, and pragmatic approach to annotation. Although the pretrained model is derived from images of common objects rather than fluorescence cytology, pretrained convolutional features often improve optimization and sample efficiency even when transferred across markedly different imaging domains. Segmentation-based approaches may provide more precise delineation of cellular boundaries, but they require substantially more detailed pixel-level annotation. Accordingly, a limitation of the present approach is that it yields bounding-box detections rather than precise cell segmentation.

The DL model achieved a precision of 69% and recall of 75% overall; however, DSE cells had substantially lower precision (57%) and recall (61%) than the other cell phenotypes. The lower performance was likely related to phenotype-specific imaging and morphological challenges. DSE cells often exhibited weaker cytoplasmic staining with less distinct cell borders, raising uncertainty in defining the cell boundaries and making localization less precise. This effect is compounded with overlapping DSE cells, as distinguishing individual cells within a cluster was more difficult for both annotation and model detection.

The DL-based object detection was significantly more reproducible than the feature extraction and ML approach. These results suggest that the ML approach, which involved hand-crafted feature extraction steps tailored to a limited set of data, was more sensitive to noise, segmentation errors, and variable imaging conditions. In contrast, the DL method provided more robust measurements, in part due to data augmentation strategies employed during training (random flips, rotation, Gaussian blur, contrast/brightness adjustments) to enhance model robustness to both morphological variation and imaging artifacts commonly encountered in cytology preparations (e.g., nonuniform cellular/nuclear staining, variability in optical focus, background noise). Image augmentations effectively expand the diversity of the training set without requiring additional manual annotations, allowing the DL model to generalize more effectively across heterogeneous samples.

The DL-based cytology test demonstrated excellent reliability and repeatability, attributes that are critical not only for distinguishing subtle cytometric differences across diagnostic categories, but also for the potential to track within-individual changes over time. High reliability ensures that measurements primarily reflect the true biological signal rather than random error, while strong repeatability minimizes variability when the same sample or patient is measured under consistent conditions. As a result, longitudinal changes can be interpreted with greater confidence as genuine shifts in disease status rather than artifacts of the measurement process. This capability is particularly important in precision lesion diagnostics, where longitudinal monitoring is based on comparing an individual’s results to their own prior values rather than to population-based references, making it essential to differentiate true temporal changes in test parameters from variability due to measurement error. Evaluation of this cytology platform for monitoring applications is ongoing in a prospective longitudinal study of patients with a prior OSCC, for recurrence, and with OPMDs harboring OED, for malignant transformation.

The POC Cyt-MF platform^10^ has the potential to deliver cytology results within minutes as compared to days for traditional labour-intensive laboratory pathology methods. Here, we presented preliminary results showing the DL model applied to data collected using the POC Cyt-MF platform, verifying that the model appropriately classified the expected cell phenotypes in the respective cytology samples (99% DSE cells in the healthy control versus 99% SR cells in the OSCC cell line). This preliminary finding suggests the potential for the DL model, which was trained with data collected using less integrated microfluidic devices and conventional fluorescent microscopes, to be translated to the integrated POC Cyt-MF platform. Additional validation of the DL model on the integrated Cyt-MF platform is in progress.

This study has limitations. Due to restrictions of the protocol, reproducibility measurements were performed by repeat testing of the same cytological sample rather than by repeat brush sampling of the same lesion. Therefore, the reliability and repeatability results only reflected analytical and post-analytical errors of the cytology test and did not account for pre-analytical errors. The oral brush cytology sampling process is subject to variability that may influence the test results, including potential sampling variability (epithelial depth and location), operator variability (differences in pressure, angle, or thoroughness of brushing), and patient-related factors that could complicate brushing (lesion location, thickness of surface keratin, bleeding, or lesion discomfort during brushing). Sampling variability may alter the relative composition of cell phenotypes within a specimen and thereby affect the test result. Further studies using repeat brush sampling of the same lesion are needed to determine the contribution of sampling variability to overall test reproducibility.

Another limitation of this study was that cellular annotations were generated by a single annotator and were not subjected to formal multi-reviewer verification or inter-annotator agreement analysis. Consequently, the consistency of the annotation process could not be formally evaluated. Future studies should incorporate annotation by multiple reviewers, ideally with expert cytopathology input and adjudication of discrepant labels.

In conclusion, by combining reliability, repeatability, and clinical relevance, this minimally invasive Cyt-MF platform holds promise as a transformative tool for early disease detection and precision monitoring in patients with OPMDs and previously diagnosed OSCC. The oral cancer numerical index (OCNI) described in this study paves the way for a new era of precision lesion diagnostics by quantitatively scoring morphologic and molecular changes for POC risk stratification and longitudinal monitoring of OPMDs. The OCNI model development and validation, clinical decision thresholds for stratifying risk, and additional diagnostic performance assessments will be detailed in a future report.

## Methods

### Study design and participants

The data used in the DL model training and validation were derived from the Grand Opportunity study, a four-site, international, prospective, non-interventional study that evaluated a laboratory-based cytology-on-a-chip system for its ability to classify oral mucosal lesions according to histopathologic diagnosis^8,9^. The study was conducted according to the Declarations of Helsinki and Istanbul and was approved by the Institutional Review Boards of participating institutions (University of Texas Health Science Center at San Antionio, HSC20070148H; University of Texas Health Science Center at Houston, HSC-DB-07–0604; University of Sheffield, 09/H1308/131; Rice University, 10–155 F). All subjects provided written informed consent.

Histopathological and brush cytological samples were collected between July 2010 and December 2012 from three groups: Group 1, subjects with OPMDs who underwent scalpel biopsy as per standard of care; Group 2, subjects with recently diagnosed OSCC; and Group 3, healthy controls without lesions. Group 1 subjects were adults with lesions ≥ 5 mm in diameter with OPMD diagnosed clinically for which a conventional scalpel biopsy was indicated. Group 2 subjects were adults with a malignant oral lesion by incisional scalpel biopsy within 45 days of the study enrollment visit, with the remaining lesion large enough to allow brushing ≥ 5 mm away from the scalpel biopsy site and the area of the lesion available for brushing ≥ 5 mm in diameter. Group 3 subjects were adults without lesions and with normal-appearing oral mucosa upon expert clinical examination (normal color, texture, and form).

Scalpel biopsy and histopathology were performed in subjects in Groups 1 and 2. Histopathological diagnoses included benign, mild OED, moderate OED, severe OED or carcinoma in situ, and malignant/OSCC^28^. Conventional OED grading is often considered subjective and lacking intra- and inter-observer reproducibility^29^. Thus, an adjudication process was developed whereby adjacent serial histologic sections were independently scored by two pathologists^8^. In the event of disagreement in scoring, a third independent pathologist reviewed both sections. If the adjudicator did not agree with either of the initial two pathologists, a third stage consensus review was conducted to establish a final diagnosis^8^.

### Study procedures

Brush cytological specimens were collected and processed as previously described^9^. Subjects underwent brush sampling of the OPMDs or site of recently diagnosed OSCC. The brush cytology sample was obtained immediately before the OPMDs underwent an incisional or excisional biopsy. Subjects with recently diagnosed OSCC were not re-biopsied. For healthy controls, brush cytology samples of normal appearing mucosa from both the lateral/ventral surface of the tongue and buccal mucosa were obtained. Brush cytology samples were collected using a Rovers Orcellex brush using mild pressure and rotating 360 degrees approximately 10–15 times to obtain the cytologic sample.

After collection, cells were harvested from the brush in culture medium, washed, cryopreserved in FBS with DMSO, frozen at − 80 °C, then later thawed, washed, fixed in formaldehyde, and stored until analysis. Before sample delivery, the cell suspension was diluted in glycerol/PBSA to improve cell distribution across the membrane and to reduce cell clumping. The microfluidic device was positioned on a robotically controlled microscope stage and connected to a peristaltic pump and injector valve. All assays contained 0.33 µM Phalloidin-AlexaFluor-647 for cytoplasmic counterstaining and 5 µM DAPI for nuclear counterstaining. The assay sequence involved priming the lab-on-a-chip devices with PBS, followed by delivery of the cell suspension, delivery of detection reagents, and a final wash step.

Images were captured automatically using a motorized fluorescence microscope at 10X magnification. A total of 25 unique image fields corresponding to a 20 mm^2 ^area were collected at three focal planes. The images at each focal plane were combined into a single, enhanced depth-of-field image using ImageJ^30^..

Repeat testing on the same samples was performed up to six times. All samples were processed using the same protocols throughout the duration of the study period.

### Deep learning model training and validation

Previously, we found that the relative proportions of the following cell phenotypes within a cytological sample demonstrated predictive value in discriminating histopathological grades^10,11^: differentiated squamous epithelial (DSE) cells, small round (SR) cells, leukocytes, and lone nuclei (LN). In this work, we trained a DL model to detect these same phenotypes and measure their proportions in each cytological sample.

The data was drawn from the prior 1053-patient GO study with > 13 million single cell images. A small subset of the available images was randomly selected representing a range of histopathological diagnoses and cell phenotypes. Images were cropped into sub-images with dimensions corresponding to the network’s input layer (e.g., 640 × 640 pixels), allowing the labeling of cells at their highest possible spatial resolution. Annotations were performed by drawing bounding boxes around each cellular object using Label Studio (version 1.18.0). Due to funding constraints, the bounding box annotations were generated by a single annotator and were not independently performed or formally verified by expert cytopathologists, and thus inter-annotator agreement was not assessed.

The training and validation sets included:

- DSE cells: 2181 instances across 434 images.
- SR cells: 3793 instances across 365 images.
- Leukocytes: 1741 instances across 298 images.
- LN: 616 instances across 229 images.

The DL object detection model was developed in Python (version 3.12.6) using the KerasCV (version 0.9.0) implementation of the YOLOv8 pre-trained model (COCO dataset, large). Training was performed using Amazon Web Services (AWS) DL AMI (Ubuntu 22.04.5 LTS) on a g4dn.xlarge EC2 instance with GPU acceleration enabled using CUDA (version 12.5.40). Additional packages used for data handling, image processing, and analysis included TensorFlow (version 2.18.1), NumPy (version 1.26.4), and OpenCV (version 4.11.0.86). Evaluation and plotting were performed using MATLAB R2025b.

Annotated images in the training set were randomly split into a ratio of 80:20 (train : validation). The model was trained using the Adam optimizer with a low learning rate (0.0001) and gradient clipping to ensure stability and prevent overfitting. During training, data augmentations including random flips, rotation, Gaussian blur, contrast, and brightness adjustments were applied exclusively to the training set to increase robustness to morphological variation and imaging artifacts. Validation was performed at the end of each epoch to evaluate model generalization. Performance metrics, including mean Average Precision (mAP), precision, and recall, were tracked to monitor convergence and guide early stopping and hyperparameter tuning. The best-performing model weights based on validation mAP were saved for future inference. Predictions from the model were post-processed using non-maximum suppression to remove redundant bounding boxes.

Accuracy was assessed on a separate hold-out set, which was prepared independently from the set used in training to ensure no data leakage. Subject-level separation ensured that images from the same subject were not included in both training and test sets. The annotated hold-out test set included:

- DSE cells: 1011 instances across 146 images.
- SR cells: 1843 instances across 116 images.
- Leukocytes: 466 instances across 97 images.
- LN: 194 instances across 83 images.

The number of images and annotated cell instances included in the training/validation and test sets was determined empirically based on the subset of fluorescence cytology images that had undergone manual annotation and met quality requirements for model development. Because annotation of individual cell phenotypes is labour-intensive, dataset size was constrained primarily by annotation feasibility rather than by a formal saturation analysis or pilot experiments. Dataset construction was therefore pragmatic and intended to provide sufficient examples for initial model development across the phenotypic classes of interest.

To support phenotype representation, images were selected so that the training/validation and test sets included examples of each annotated cell class. Selection was not based solely on image count, but also on the presence of annotated instances from the different phenotype categories. Where class imbalance existed, the dataset split was designed to retain representation of less frequent phenotypes in both subsets as much as possible within the available annotated dataset.

The accuracy of the DL model in detecting each cell phenotype was assessed using average precision and average recall at various intersection over union (IoU) criteria (0.5:0.95, ≥ 0.5, and ≥ 0.75). Precision and recall were assessed for all cells, all cells stratified by size, and for each cell phenotype individually.

### Statistical analysis

Descriptive statistics (mean ± SD or n [%]) were assessed for healthy controls, subjects with benign lesions, and subjects with OED or OSCC. Subjects with benign lesions were compared to subjects with OED and OSCC, with continuous data analyzed using independent two-sample t-test and proportions analyzed using chi-squared test, with *p* = 0.05 considered statistically significant.

The following cytological test parameters were calculated: relative percentages of DSE cells, SR cells, and leukocytes; approximate median cell diameter of DSE and SR cells (i.e., square root of bounding box area converted from pixels to µm); and the oral cancer numerical index (OCNI)^10,11^, a score from 0 to 100 that represents the probability of OED or OSCC (predictors included age, sex, tobacco history, lesion color, lesion size, lesion appearance, presence of multiple lesions, DSE cells, and SR cells).

Distributions of cell phenotype data were compared across histopathological diagnoses. Spearman rank correlation coefficients assessed the monotonic relationship, and Kruskal-Wallis compared median values across histopathological diagnoses.

Diagnostic performance by area under the receiver operating characteristic curve (AUROC) was evaluated for cell phenotype data in discriminating the following dichotomous splits of histopathological diagnoses: benign versus OED/OSCC; benign and mild OED versus moderate OED through OSCC; benign through moderate OED versus severe OED and OSCC; benign versus OSCC; and healthy control versus OSCC.

Test reliability and repeatability were evaluated for each paired test (up to six tests) for all subjects and by histopathological grading. Intraclass correlation coefficients (ICC) were calculated using single rater/measurement, two-way mixed effects model for absolute agreement among measurements with one-sided test for the lower acceptable limit of 0.75 (*p*< 0.05). ICC values indicate the degree of reliability of the measurements, with values < 0.5 considered poor, 0.5–0.74 moderate, 0.75–0.90 good, and > 0.90 excellent reliability^31^. Minimum detectable difference (MDD)^32–34^, interpreted as the smallest detectable difference in value of a test parameter before and after experimental manipulation (e.g., treatment) which is larger than would be expected by chance, was calculated for each test parameter. Within-sample repeatability was assessed by %CV.

Test reliability and repeatability were compared to an earlier version of the cell phenotype algorithm that used a feature extraction and ML approach^10,11^. In this previous version, the image analysis software CellProfiler^35^ identified individual cell objects, defined nuclear and cytoplasmic boundaries, obtained fluorescence intensity measurements for red (F-actin) and blue (DNA) spectral channels, and defined morphometric parameters. The *k*-Nearest Neighbor algorithms were trained on a subset of the 144 extracted features. The same within-sample reliability and repeatability analysis was performed for the 506 subjects with complete cytology data analyzed with this method.

All statistical analyses were performed using MATLAB R2025b.

### Evaluation of the POC Cyt-MF platform

To demonstrate proof of concept of the DL model on data collected with the POC Cyt-MF platform^10^, two assays were performed using the integrated platform, including a healthy control sample and a sample with OSCC cells (BICR 56 cell line [RRID: CVCL_2313] from human female tongue OSCC grown in 2D cell culture as monolayers). Cytology test parameters were compared between the two examples.

## Supplementary Information

Below is the link to the electronic supplementary material.

Supplementary Material 1

## Data Availability

The data tables analyzed for the current study can be made available from the corresponding author on reasonable request. Individual patient data will not be shared due to privacy issues.

## Code availability

The source code, trained model weights, and full analysis pipeline are not publicly available because they constitute proprietary components of a technology under active commercial development. The manuscript provides detailed descriptions of the software environment, model architecture, data processing workflow, and statistical analysis details to enable evaluation of the methodological approach as thoroughly as possible given the current restrictions. Requests for additional technical information or limited access arrangements may be considered for academic, independent research, or regulatory review purposes under appropriate confidentiality agreements.
