# Supplementary material for: Deep learning single-cell analysis for cytologic evaluation of oral potentially malignant disorders
Source: Sci Rep. 2026 Jul 13;16:21741. doi: 10.1038/s41598-026-47538-y (PMC13357536; doi:10.1038/s41598-026-47538-y)
Supplement: Supplementary file 1 — Supplementary Material 1 [file 41598_2026_47538_MOESM1_ESM.docx]

# Supplemental Materials


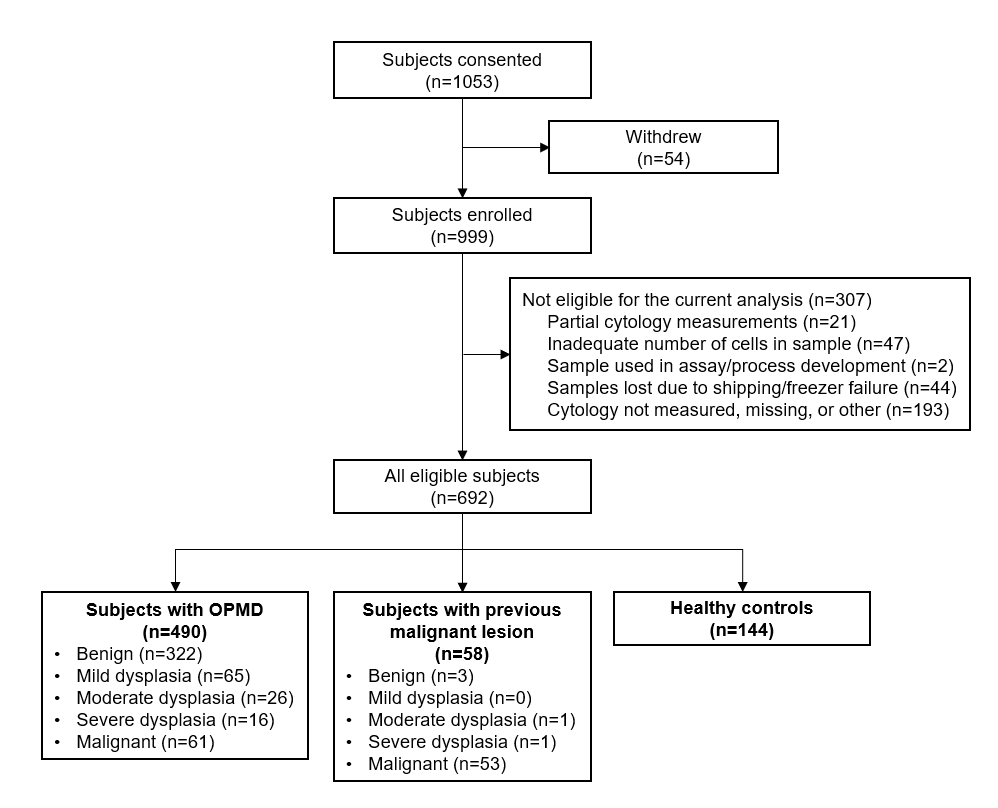


**Figure S1.** Grand Opportunity Study subject disposition.

**Table S1.** Average precision and average recall for identifying cell phenotypes.

| **Class** | **IoU** | **Average Precision** | **Average Recall** |
| --- | --- | --- | --- |
| All cells | 0.5:0.95 | 69.0% | 74.7% |
|  | ≥0.5 | 73.1% | - |
|  | ≥0.75 | 71.6% | - |
| DSE cells | 0.5:0.95 | 57.2% | 61.2% |
|  | ≥0.5 | 63.2% | - |
|  | ≥0.75 | 60.8% | - |
| SR cells | 0.5:0.95 | 72.0% | 73.1% |
|  | ≥0.5 | 74.9% | - |
|  | ≥0.75 | 73.9% | - |
| Leukocytes | 0.5:0.95 | 78.8% | 80.5% |
|  | ≥0.5 | 81.6% | - |
|  | ≥0.75 | 80.4% | - |
| Lone nuclei | 0.5:0.95 | 68.2% | 84.0% |
|  | ≥0.5 | 72.7% | - |
|  | ≥0.75 | 71.2% | - |

Abbreviations: IoU, intersection over union; DSE, differentiated squamous epithelial cells; SR, small round parabasal-like cells.

**Table S2.** Diagnostic performance of cytology test parameters relative to histopathology.

|  | **AUROC (95% CI)** |
| --- | --- |
| Benign (n=325) vs. Mild–Malignant (n=223) |  |
| DSE cells | 0.73 (0.68 – 0.77) |
| SR cells | 0.73 (0.69 – 0.78) |
| Leukocytes | 0.66 (0.61 – 0.71) |
| Median cell diameter | 0.71 (0.67 – 0.76) |
| OCNI | 0.78 (0.74 – 0.82) |
| Benign–Mild (n=390) vs. Moderate–Malignant (n=158) |  |
| DSE cells | 0.81 (0.77 – 0.85) |
| SR cells | 0.77 (0.73 – 0.82) |
| Leukocytes | 0.75 (0.70 – 0.80) |
| Median cell diameter | 0.79 (0.74 – 0.83) |
| OCNI | 0.87 (0.84 – 0.91) |
| Benign–Moderate (n=417) vs. Severe–Malignant (n=131) |  |
| DSE cells | 0.85 (0.81 – 0.89) |
| SR cells | 0.78 (0.74 – 0.82) |
| Leukocytes | 0.81 (0.76 – 0.86) |
| Median cell diameter | 0.81 (0.77 – 0.85) |
| OCNI | 0.90 (0.87 – 0.93) |
| Benign (n=325) vs. Malignant (n=114) |  |
| DSE cells | 0.87 (0.84 – 0.91) |
| SR cells | 0.81 (0.77 – 0.85) |
| Leukocytes | 0.83 (0.77 – 0.87) |
| Median cell diameter | 0.84 (0.80 – 0.88) |
| OCNI | 0.92 (0.89 – 0.94) |
| Control (n=144) vs. Malignant (n=114) |  |
| DSE cells | 0.99 (0.98 – 1.00) |
| SR cells | 0.97 (0.96 – 0.99) |
| Leukocytes | 0.98 (0.95 – 0.99) |
| Median cell diameter | 0.96 (0.93 – 0.98) |
| OCNI | 0.99 (0.99 – 1.00) |

Abbreviations: AUROC, area under the receiver operating characteristic curve; CI, confidence interval; DSE, differentiated squamous epithelial cells; SR, small round parabasal-like cells; OCNI, oral cancer numerical index.

**Table S3.** Median (IQR) values of cytology test parameters by histopathological diagnosis.

| **Parameter** | **Control**  n=144 | **Benign**  n=325 | **Mild**  n=65 | **Moderate**  n=27 | **Severe**  n=17 | **OSCC**  n=114 |
| --- | --- | --- | --- | --- | --- | --- |
| DSE Cells (%) | 96.5  (95.2-97.5) | 90.0  (78.5-94.4) | 87.2  (78.2-93.8) | 82.4  (68.5-92.5) | 74.7  (57.5-89.5) | 34.9  (14.3-75.0) |
| SR Cells (%) | 2.7  (2.0-3.8) | 6.0  (3.5-12.0) | 10.6  (4.6-16.5) | 13.9  (6.2-23.8) | 11.8  (8.8-22.6) | 18.7  (14.2-28.0) |
| Leukocytes (%) | 0.7  (0.5-1.0) | 2.2  (1.2-5.2) | 2.1  (1.1-3.5) | 2.1  (0.9-5.5) | 3.6  (1.8-9.8) | 35.2  (6.6-58.2) |
| Lone nuclei (%) | 13.4  (6.5-26.5) | 21.0  (11.9-31.8) | 14.8  (9.6-24.6) | 23.8  (11.4-35.7) | 18.0  (11.6-24.2) | 14.6  (8.4-22.4) |
| Median cell diameter (µm) | 72.4  (70.3-74.9) | 69.1  (63.6-72.8) | 68.2  (63.9-71.6) | 65.8  (59.8-69.4) | 64.9  (61.1-67.4) | 55.9  (43.8-64.3) |
| OCNI | 14.8  (11.0-18.9) | 26.5  (18.9-39.2) | 29.7  (22.1-41.4) | 43.0  (37.7-55.5) | 56.1  (35.4-66.4) | 74.6  (57.5-85.3) |

Abbreviations: Mild, mild dysplasia; Moderate, moderate dysplasia; Severe, severe dysplasia & carcinoma *in situ*; OSCC, oral squamous cell carcinoma; DSE, differentiated squamous epithelial cells; SR, small round parabasal-like cells; OCNI, oral cancer numerical index.

**Table S4**. Summary of within-sample repeat testing.

| **# of repeat tests** | **# of subjects** |
| --- | --- |
| 6 | 604 |
| 5 | 68 |
| 4 | 6 |
| 3 | 13 |
| 2 | 0 |
| 1 | 1 |
|  |  |
| Total subjects | 692 |
| Total tests | 4028 |

**Table S5.** Within-sample reliability and repeatability of the cytological assay across all subjects, and by histopathologic diagnosis, with each sample measurement repeated up to 6 times.

| **Parameter** | **Mean** | **SD of within-subject differences** | **%CV** | **MDD** | **ICC (95% CI)** | **p value** |
| --- | --- | --- | --- | --- | --- | --- |
| All Subjects (n=692) |  |  |  |  |  |  |
| DSE Cells (%) | 78.4 | 7.0 | 7.3% | 2.82 | 0.98 (0.97-0.98) | **<0.0001** |
| SR Cells (%) | 11.4 | 6.4 | 36.3% | 6.49 | 0.87 (0.82-0.89) | **<0.0001** |
| Leukocytes (%) | 10.2 | 5.7 | 47.1% | 2.76 | 0.97 (0.96-0.97) | **<0.0001** |
| Median cell diameter (µm) | 66.1 | 3.8 | 3.4% | 2.12 | 0.96 (0.95-0.97) | **<0.0001** |
| OCNI | 35.7 | 5.5 | 8.2% | 2.16 | 0.98 (0.97-0.98) | **<0.0001** |
| Healthy controls (n=144) |  |  |  |  |  |  |
| DSE Cells (%) | 95.6 | 2.7 | 1.7% | 4.36 | 0.66 (0.57-0.74) | 0.9879 |
| SR Cells (%) | 3.5 | 2.3 | 42.0% | 3.73 | 0.65 (0.56-0.73) | 0.9937 |
| Leukocytes (%) | 0.9 | 0.9 | 53.7% | 1.77 | 0.53 (0.45-0.60) | 1.0000 |
| Median cell diameter (µm) | 72.4 | 3.0 | 2.6% | 4.18 | 0.74 (0.67-0.80) | 0.6096 |
| OCNI | 16.4 | 1.7 | 5.3% | 0.95 | 0.96 (0.95-0.97) | **<0.0001** |
| Benign (n=325) |  |  |  |  |  |  |
| DSE Cells (%) | 82.3 | 7.9 | 6.4% | 6.94 | 0.90 (0.88-0.92) | **<0.0001** |
| SR Cells (%) | 10.2 | 6.2 | 37.6% | 7.51 | 0.81 (0.76-0.85) | **0.0081** |
| Leukocytes (%) | 7.6 | 5.1 | 50.8% | 3.9 | 0.92 (0.91-0.93) | **<0.0001** |
| Median cell diameter (µm) | 67.6 | 4.2 | 3.4% | 4.19 | 0.87 (0.84-0.89) | **<0.0001** |
| OCNI | 31.8 | 6.1 | 9.6% | 4.45 | 0.93 (0.91-0.94) | **<0.0001** |
| OED (n=109) |  |  |  |  |  |  |
| DSE Cells (%) | 80.8 | 8.2 | 7.0% | 8.86 | 0.85 (0.80-0.89) | **0.0004** |
| SR Cells (%) | 14.5 | 6.6 | 31.6% | 7.15 | 0.85 (0.77-0.90) | **0.0074** |
| Leukocytes (%) | 4.7 | 4.1 | 49.5% | 4.89 | 0.82 (0.77-0.86) | **0.0027** |
| Median cell diameter (µm) | 66.4 | 3.6 | 3.2% | 3.36 | 0.89 (0.86-0.92) | **<0.0001** |
| OCNI | 38.7 | 6.9 | 10.6% | 5.51 | 0.92 (0.88-0.94) | **<0.0001** |
| Malignant (n=114) |  |  |  |  |  |  |
| DSE Cells (%) | 42.6 | 7.1 | 17.6% | 1.9 | 0.99 (0.99-0.99) | **<0.0001** |
| SR Cells (%) | 22.4 | 9.0 | 30.2% | 10.99 | 0.81 (0.67-0.88) | 0.1797 |
| Leukocytes (%) | 35.0 | 9.7 | 25.5% | 6.89 | 0.93 (0.89-0.96) | **<0.0001** |
| Median cell diameter (µm) | 53.6 | 4.0 | 4.7% | 0.72 | 1.00 (0.99-1.00) | **<0.0001** |
| OCNI | 69.1 | 5.3 | 5.5% | 3.27 | 0.95 (0.93-0.96) | **<0.0001** |

Abbreviations: ICC, intra-class correlation coefficient; %CV, coefficient of variation; MDD, minimum detectable difference; CI, confidence interval; DSE, differentiated squamous epithelial cells; SR, small round parabasal-like cells; OCNI, oral cancer numerical index; OED, oral epithelial dysplasia (mild, moderate, severe and carcinoma *in situ*).

**Table S6.** Within-sample reliability and repeatability of results derived from CellProfiler and machine learning-based cell phenotype model (McRae et al. 2020).

| **Parameter** | **Mean** | **SD of within-subject differences** | **%CV** | **MDD** | **ICC (95% CI)** | **p value** |
| --- | --- | --- | --- | --- | --- | --- |
| All Subjects (n=506) |  |  |  |  |  |  |
| DSE Cells (%) | 84.4 | 15.0 | 12.9% | 21.38 | 0.74 (0.69-0.78) | 0.0782 |
| SR Cells (%) | 7.5 | 12.0 | 85.3% | 25.44 | 0.42 (0.37-0.46) | 1.0000 |
| Leukocytes (%) | 8.5 | 8.8 | 75.2% | 10.73 | 0.81 (0.75-0.85) | **0.0006** |
| OCNI | 32.7 | 8.8 | 20.6% | 6.75 | 0.92 (0.90-0.94) | **<0.0001** |
| Healthy controls (n=125) |  |  |  |  |  |  |
| DSE Cells (%) | 93.0 | 12.3 | 7.3% | 30.47 | 0.20 (0.13-0.28) | 1.0000 |
| SR Cells (%) | 4.7 | 10.1 | 86.6% | 25.6 | 0.16 (0.10-0.23) | 1.0000 |
| Leukocytes (%) | 2.9 | 4.9 | 74.7% | 11.81 | 0.24 (0.15-0.34) | 1.0000 |
| OCNI | 20.9 | 8.0 | 24.7% | 11.78 | 0.72 (0.61-0.80) | 0.3560 |
| Benign (n=253) |  |  |  |  |  |  |
| DSE Cells (%) | 87.6 | 13.5 | 10.5% | 21.39 | 0.68 (0.61-0.73) | 0.7981 |
| SR Cells (%) | 5.9 | 10.3 | 87.9% | 24.27 | 0.28 (0.22-0.34) | 1.0000 |
| Leukocytes (%) | 7.0 | 8.2 | 78.6% | 10.78 | 0.78 (0.70-0.83) | **0.0217** |
| OCNI | 27.1 | 8.4 | 21.8% | 7.52 | 0.90 (0.87-0.92) | **<0.0001** |
| OED (n=63) |  |  |  |  |  |  |
| DSE Cells (%) | 84.2 | 15.2 | 12.0% | 26.17 | 0.61 (0.50-0.72) | 0.9451 |
| SR Cells (%) | 9.0 | 13.2 | 84.8% | 28.2 | 0.41 (0.30-0.53) | 1.0000 |
| Leukocytes (%) | 7.1 | 8.1 | 79.5% | 13.39 | 0.64 (0.47-0.77) | 0.8024 |
| OCNI | 38.9 | 9.9 | 18.3% | 9.61 | 0.88 (0.81-0.92) | **<0.0001** |
| Malignant (n=65) |  |  |  |  |  |  |
| DSE Cells (%) | 56.3 | 22.7 | 33.9% | 34.87 | 0.69 (0.58-0.79) | 0.5479 |
| SR Cells (%) | 17.7 | 18.5 | 72.8% | 39.27 | 0.41 (0.31-0.53) | 1.0000 |
| Leukocytes (%) | 26.1 | 15.0 | 58.3% | 18.84 | 0.79 (0.71-0.86) | **0.0166** |
| OCNI | 71.3 | 10.4 | 10.5% | 10.73 | 0.86 (0.79-0.91) | **0.0001** |

Abbreviations: ICC, intra-class correlation coefficient; %CV, coefficient of variation; MDD, minimum detectable difference; CI, confidence interval; DSE, differentiated squamous epithelial cells; SR, small round parabasal-like cells; OCNI, oral cancer numerical index.
